# Effective therapeutic interventions for Australian adolescents using alcohol and/or other drugs: a scoping review

**DOI:** 10.1186/s13033-020-00425-z

**Published:** 2020-12-07

**Authors:** Jennifer Martin, Marg Liddell, Susan Roberts, Emily Greenwood

**Affiliations:** 1 Social Innovation Research Institute, Faculty of Health Arts and Design, Swinburne University, Swinburne University of Technology, Office AS430 L Internal Mail H31, PO Box 218, Hawthorn, VIC 3122 Australia; 2 Social and Global Studies Centre, RMIT University, GPO Box 2476, Melbourne, VIC 3001 Australia; 3 Swinburne University of Technology, Swinburne Library C/-AS430 L Internal Mail H31, PO Box 218, Hawthorn, VIC 3122 Australia; 4 Swinburne University of Technology, Office: c/-AS430 l Internal Mail H31, PO Box 218, Hawthorn, VIC 3122 Australia

**Keywords:** Adolescent, Alcohol and other drugs, Substance use, Therapeutic interventions

## Abstract

**Background:**

There are a variety of residential and community service models of therapeutic interventions for people using substances. The focus of much of the currently available research is on adult populations with relatively little known about effective therapeutic interventions for adolescents. The aim of this paper is to identify the most effective therapeutic interventions for Australian adolescents using substances by conducting a systematic scoping literature review.

**Methods:**

We followed the PRISMA guidelines to conduct a systematic scoping review that included searches of nine electronic databases (EMBASE, MEDLINE, EBSCO Host, APA PsycNet, SocIndex, Social Science Abstracts, Proquest Central Informit) and grey literature searches of government department and alcohol and other drugs peak body and service provider websites, Google Scholar and the Cochrane library.

**Results:**

A total of 21 studies were identified. These studies included biological, psychological, social and technological therapeutic interventions targeted at different population groups and different substances. The review findings are limited and should considered with caution due to the inability to disaggregate the combinations of interventions provided and the low quality of most of the studies included.

**Conclusions:**

This scoping review highlights the paucity of quality research on effective therapeutic interventions for Australian adolescents using substances. This is primarily due to the available studies not controlling for all of the therapeutic interventions provided. While there is an evidence-base for some of these interventions, others such as encounter groups and journaling require further and more substantive research for use with adolescents. This is necessary to enable informed service design and delivery decision-making and fiscal accountability.

## Background

Substance use disorders typically have their onset in adolescence so effective treatments are critical for this age group. Adolescents are particularly vulnerable to detrimental physical, psychological and social impacts of drug use. These impacts include significant harm to the developing brain and functional capacity; poor physical, mental, sexual and reproductive health; lowered educational achievement and violence [1, 2]. In particular, executive brain processes for the achievement of higher order goals are developing during adolescence. This is in the context of increased brain plasticity, sensation seeking and vulnerability to the development of psychopathology. Adolescence can be an uncertain time given the major changes that are occurring physically, psychologically and socially. As a result, interventions suited to adults are not necessarily appropriate for adolescents due to their reduced reasoning capacity and the developmental tasks associated with adolescence including identity and reputation formation and increased autonomy, independence, self-esteem and confidence [3].

The focus of this paper is on how to best respond therapeutically to problematic use of substances by adolescents. There is no agreed universal definition of substance abuse and the terminology used varies and includes terms such as substance use, abuse, misuse, harmful use, hazardous use, substance dependence and addiction. At times, the definitions of these terms are ambiguous creating confusion [4]. For instance, in the latest edition of the Diagnostic and Statistical Manual (DSM5) the American Psychiatric Association uses the term substance abuse [5]. However, in the International Classification of Diseases (ICD-11) the preferred terminology of the World Health Organisation (WHO) is “harmful use” or “hazardous use”. For the purpose of this study substance use is the preferred term and it encompasses all types of drug use [6]. The WHO (2020) considers term such as abuse to be disapproving language with the Australian government adopting the language of substance use to support a non-judgmental approach to assist people to disclose usage in a supported environment and to seek appropriate assistance [7].

Therapeutic intervention was defined as an intervention to enhance an adolescent’s wellbeing. This may be within a context of voluntary help-seeking by the adolescent or in situations where help has not been actively sought by the adolescent and be provided in person or online [8].

Various explanatory models of drug use exist in alcohol and other drug services including disease, moral and social approaches. Service providers are from diverse backgrounds and use varied treatment approaches that are provided in residential and community settings [9].

The context of Australia was chosen for this scoping review by the funding body who is a not-for-profit provider of alcohol and other drug rehabilitation services to Australian adolescents. This organisation wanted to know what are considered to the most effective therapeutic interventions for Australian adolescents 12 to 17 years of age who are at high risk of drug-related harms due to their substance use. They were particularly interested in what works in the Australian context noting the political, economic and social differences globally. This approach is supported by the joint United Nations/ World Health Organisation *SAFER: A Safer World Free from Alcohol Related Harms* (2019) initiative that focuses on in-country action within a global context [10]. The focus on Australia was with the understanding that these Australian studies were located within the international literature. It is anticipated that other countries can also learn from a closer look at the Australian experience given that the types of services offered in Australia are similar to those in other countries around the world including motivational interviewing (MI), cognitive behaviour therapy (CBT) combined with other psycho-social, education and recreation interventions.

## Methods

We used scoping review methodology to achieve our aim of providing a broad rapid overview of a research topic and to identify common themes, issues and gaps in the literature on the most effective therapeutic interventions with a minority of adolescents who have problematic substance use [11]. The review process followed the Cochrane approach and included: (1) selection of search terms and databases, (2) search of refereed and grey literature and hand searching, (3) title and abstract screening, (4) full text review and (5) quality appraisals [12]. Scoping reviews include all relevant literature with the refereed literature consisting of both single studies as well as systematic reviews (Sucharew & Macaluso) [13]. The literature was mapped, categorized and described to provide evidence of effective therapeutic intervention outcomes.

### Research question and definitions

The aim was to answer the study question: ‘What does the evidence in the Australian literature tell us about what are the most effective therapeutic interventions for adolescents, aged 12 to 17 years, using alcohol and/or other drugs?’ The current literature review expands the scope of previous reviews by looking at all reported effective therapeutic interventions for all substance use including both illicit and licit substances (tobacco, pharmaceuticals and illicit drugs) [14]. To the best of the authors’ knowledge this is the first scoping review to consider the combination of all substances, misuse and abuse and effective therapeutic interventions for adolescents.

### Selection of studies

The online search databases MEDLINE, EMBASE, APA PsycNet, EBSCO Host, Social Science Abstracts, SocIndex, Informit, Proquest Central, the Cochrane library databases and Google Scholar were chosen due to breadth and relevance to the topic. Targeted browsing was conducted of publications on national, state and territory government and non-government websites. Hand searching of bibliographies and alcohol and other drug training notes was also undertaken. Search terms included ‘adolescent’ or ‘youth’ or ‘teenage’ or ‘young’ and ‘drug’ or ‘alcohol’ or ‘substance abuse’ and ‘recovery program’, or ‘drug education’ or ‘intervention’, or ‘treatment’ and ‘Australia’. The search date range was set from 2010 to 2018 (current) with language set as English. This search time-frame was chosen so as to build upon knowledge gained from previous systematic reviews on this topic.

Types of studies included in the review were meta-analyses and literature reviews, controlled experimental studies, before-and-after studies with no control group, observational studies, cohort studies and qualitative studies. The studies needed to provide evidence of the effectiveness of specific therapeutic interventions delivered to adolescents using alcohol and/or other drugs.

### Review of studies

Two authors independently checked all of the studies by title, abstract and full text with a third author available for moderation to reduce researcher bias. The exclusion criteria at the initial screening stage resulted in the removal of papers that were (1) duplicates, (2) not an Australian study, (3) a more recent version was available, (4) published or produced before 2010, (5) inadequate reporting of methods, (6) not specific to adolescents (7) not a therapeutic intervention or (8) not specific to alcohol and other drugs. Duplicate removal included review of the individual studies and studies included in the systematic reviews.

### Data analysis

Thematic analysis was used to interpret and categorise the data into therapeutic intervention categories according to intervention type and therapeutic outcomes [15]. The data were analysed and reported according to study design, year of study, type and length of therapeutic interventions, sample size, inclusion criteria, age of participants, gender and therapeutic outcomes. Two authors categorised the data independently and then jointly to present an accurate representation of the entire dataset. Multi-level synthesis was used to integrate the quantitative and qualitative study data [16].

### Assessment of bias

Quality assessments were conducted on all of the included studies to evaluate methodological strength and risk of bias, using the recommended validated tools suited to the particular type of study. The *Cochrane Collaboration’s Tool* was used to assess controlled trials [17]; the *Critical Appraisal Skills Program (CASP) Qualitative Checklist* was used to assess qualitative studies including aims, methodology, design, recruitment, data collection, relationship between researcher/s and participants, ethical issues, data analysis, findings and significance [18], and the *Study Quality Assessment Tools* from the National Heart, Lung and Blood Institute were used to assess systematic reviews and meta-analyses, cross-sectional and observation cohort studies [19]. Systematic reviews were assessed on the adequacy of the formulation of the research question, clarity of inclusion and exclusion criteria, search strategies, independent review by two or more researchers of abstracts and full-text, internal validity, listing of included studies and key features, and publication bias [19].

## Results

**Fig. 1:**
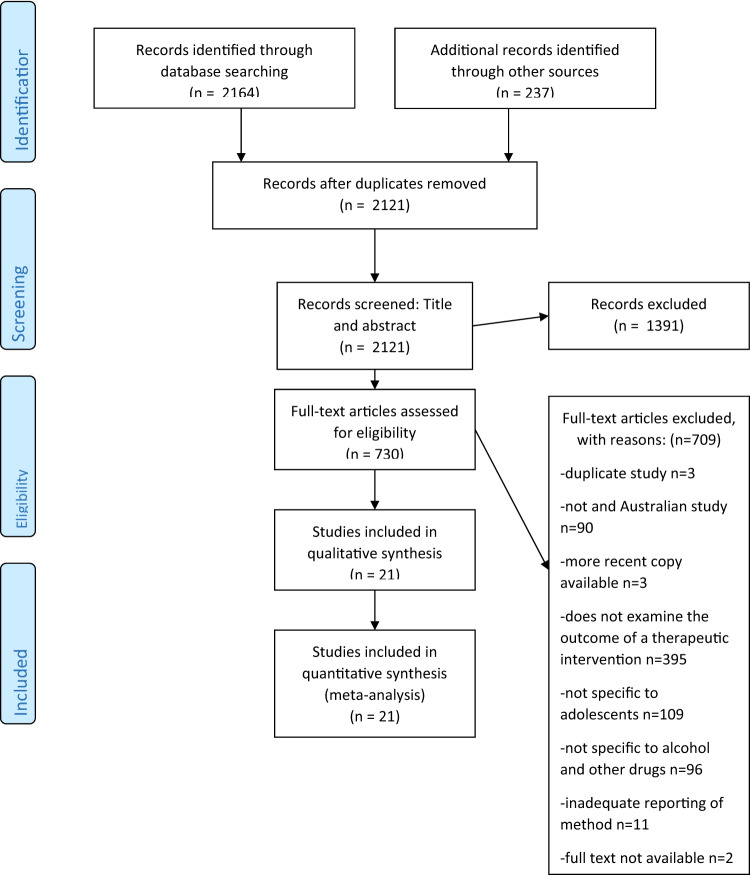
PRISMA Flow Diagram [36] search strategy results

A total of 21 studies was included comprising 3,493 participants (excluding the literature review samples). Due to only studies on effective therapeutic interventions being included some substances, such as tobacco, were not present in the study findings. The search strategy revealed 2121 texts for the initial screening of title and abstract with 730 articles progressing to full text review (see Fig. 1).

The final studies included were 17 experimental studies and 4 non-experimental studies. Most studies provided multiple interventions but did not evaluate the efficacy of all of these, thereby restricting the attribution of improved outcomes to a specific intervention. Studies were grouped according to focus of the intervention as (1) individual [20–26, 29–41]; (2) family [20, 22, 25–28]; (3) organisational [20–22, 25–28, 34, 39, 40] and (4) multiple interventions [20–22, 25, 26, 35, 40]. The following interventions were reported as producing effective therapeutic outcomes with further details of all studies provided in Table 1.

**Table 1:** Australian evidence on therapeutic interventions with adolescents with alcohol and other drug issues

First author, year of publication	Study design, year of study	Intervention(s), length of treatment	Sample	Eligibility	Age range, years (%m male)	Data collection	Outcome/effect	Risk of bias
Albertella et al. (2012)	Before-after study with no control group January 2002-July 2010	Residential substance use program Individual counselling, family counselling, living skills training, vocational/educational training, journaling and recreational activities Continuing care in the community Up to 3 month stay	Sample size: 132	14–18 years old Cannabis primary drug Completed ≥ 30 days of treatment	14–18 years (77%m)	Pre-treatment 3-month follow-up	Reduced anxiety, paranoia and interpersonal sensitivity associated with reduced cannabis use on follow-up	Low
Bushmob (2014)	Observational study/Case study/Evaluation 2009	Bush adventure therapy, counselling, individual therapy and continuing care 5-day horse trek	Sample size: 65	6–80 years old young people with substance abuse issues, family and community members	6–80 years (%m not stated)	Observations during trek	Reduced substance use and self-harm, and improved wellbeing	High
Calabria et al. (2011)	Systematic review 2005–2009	Individual intervention studies on young people experiencing alcohol-related harm	9 studies	Published in English Intervention studies	*Across studies:* 11–25 years, (17–90%m)	10 electronic databases, reference lists	None of the included studies had consistently strong methodology Cognitive behaviour therapy (CBT), family therapy and community reinforcement appeared to be the more successful approaches	Moderate
Calabria et al. (2012)	Systematic review 2003–2010	Family-based interventions for alcohol misuse and alcohol-related harm in Indigenous communities	19 studies	Published in English Family-based interventions	*Across studies:* 12–78 years Targeting problem drinkers: (17–100%m) Targeting family members (0–31%m)	11 electronic databases, reference lists	18 family-based interventions yielded a positive effect in Decreased alcohol consumption Improved individual and family coping and functioning	High
Doran et al. (2017)	Evidence check rapid literature review January 2007-March 2017	Community, school-based and therapeutic interventions targeted at Aboriginal youth at risk of AOD-related harm	52 studies	Published in English Indigenous young people Alcohol and other drugs (AOD) interventions	*Across studies:* Participant age range and %m not stated	9 electronic databases, consultation with experts	Successful AOD interventions for Indigenous young people require strong community and cultural engagement and support that is multi-faceted and flexible, addressing both individual, community and systemic issues	Moderate
Foster et al. (2010)	Participant observation/ ethnographic study 2006	Residential therapeutic community: journaling encounter groups, vocational education Average length of stay: 40 days Program length: 3 months	Sample size: 19	14–18 years old substance dependence	14–18 years (68%m)	4 months participant observation and informal interviews	Tailored recreation, art therapy and vocational education preferred Negative reactions to journaling and encounter group sessions	Moderate
Green et al. (2013)	Qualitative study 2009–2010	The function of relationships in youth-focused AOD service: outreach, day programs, residential withdrawal and residential rehabilitation Primary drugs of concern: cannabis alcohol, and heroin Length of treatment: Residential withdrawal: up to 2 weeks) Residential rehabilitation: up to 6 months	Sample size: 42	Young people aged 15–22 years who were clients Stable in AOD use and mental health Ability to reflect on experiences Excluded if recent experience of traumatic events or in emotional distress	15–22 years (52.4%m)	Semi-structured interviews	Validating relationships with family, peers and service providers are key to success	Low
Hides et al. (2010)	Before-after study with no control group Year not stated	Self-help for alcohol/other drug use and depression – for young people 10 sessions of individual integrated CBT treatment (incorporating motivational interviewing (MI) and mindfulness) Intervention: Up to 20 weeks	Sample size: 60	15–25 years old Diagnosis of Major Depressive Disorder and Substance Use Disorder or risky alcohol/drug use English speaking Excluded if using antidepressants within the past 30 days and/or a current/past history of psychosis	15–25 years (56.7%m)	Assessment at baseline, mid-treatment (10 weeks), post-treatment (20 weeks) and 6 months follow-up (44 weeks)	Integrated CBT treatment associated with significant improvements in depression, anxiety, cognition, substance use and coping skills at follow-up	Moderate
Hides et al. (2011)	Non-RCT (parallel group) 2006–2008	Standard care (SC): Case management and brief MI Intervention: SC plus CBT and MI Timeframe: Up to 12 weeks Substance misuse: mainly alcohol and cannabis	Sample size: 106 SC (n = 28) SC + CBT/MI (n = 60)	16–25 years old Comorbid depression and substance misuse Accessing treatment at youth AOD service English speaking No past/current psychosis	16–25 years (63%m)	Assessment at baseline, 3 months and 6 months	SC + CBT/MI group showed significant reductions in depression and cannabis use and increased social contact and motivation to change at 3-month follow-up compared to the SC group Both groups achieved significant improvements in functioning and quality of life at 6 months follow-up	Moderate
Hides et al. (2013)	RCT (parallel group), Year not stated	Assessment/Feedback Information (AFI) group: 1 session brief assessment, general assessment feedback Brief Motivational Interviewing (BMI) intervention group: 2–3 sessions of BMI with personalised assessment feedback, psychoeducation and brief coping skills training	Sample size: 61	16–25 years old Accessing a specialist youth mental health primary care service using cannabis and/or alcohol English speaking No past/current psychosis	16–25 years (55.7%m)	Assessment at baseline, 1, 3 and 6 months	BMI more effective in reducing alcohol use and psychological distress and achieving a more rapid reduction in cannabis use than AFI. No significant differences in both groups for psychological distress at 3 months. Reduced levels of psychological distress in BMI group at 6 months	Low
Hides et al. (2018)	RCT (parallel group), Year not stated	Mobile app informed by motivational interviewing that creates a virtual experience to increase alcohol knowledge and reduce alcohol use in young app users Duration of intervention: not specified	Sample size: 197 Immediate access group (n = 96) 1-month delayed access control group (n = 101)	16–25 years old Australian residents Consume alcohol at least monthly Own an iPhone	16–25 years (22.3%m)	Assessment at baseline, 1,2,3, and 6 months	No significant differences between the immediate and delayed access groups in alcohol use and alcohol related harm	Low
Hilferty et al. (2015)	Mixed-methods evaluation 2013 to 2014	Primary intervention: focused psychological strategies provided by headspace Comparison groups for binge drinking and cannabis use: 12–17 year-olds (n = 1,686) via survey Intervention: 5 sessions average	headspace datasets (n = 45,000) Survey participants: headspace treatment (n = 1515) No treatment (n = 4774)	12–25 years old receiving headspace services	12–25 years (%m not stated)	Comparison of young people attending headspace and young people not attending headspace	Significant small positive improvement in reduced psychological distress for the headspace group compared with the other treatment and no treatment groups over time. Reduced binge drinking highest in other treatment group. No significant differences between groups in cannabis use	Moderate
Knight et al. (2017)	Systematic review 2009–2014	Interventions including education, mentoring, recreation, information provision and counselling targeting multiple risk factors	13 studies	12–24 years with multiple risk factors	*Across studies:* 12–24 years (0–94%m)	7 electronic databases and grey literature	Most studies on single risk factors. Half of the studies were methodologically weak	High
Maclean et al. (2012)	Systematic review 1980–2010	Psychosocial therapeutic interventions for volatile substance use (VSU)	19 studies	Published in English Substance use, health or welfare outcome data for therapeutic interventions addressing volatile substance use	*Across studies:* 10–32 years (%m not stated)	Electronic databases and grey literature	Clear conclusions for VSU psychological treatment are not supported	High
Mission Australia (2011)	Before-after study with no control group 2005–2009	Residential rehabilitation: Counselling, case management, vocational education and training, sport and recreation, support for family communication and aftercare Length of treatment: Residential treatment: up to 3 months Aftercare: up to 6 months	Sample size: 399 participants in program Aftercare cohort: n = 160	16–24 years Experiencing co-morbid conditions of drug addiction and mental illness	16–24 years (72%m)	Assessment at baseline, 3 months and 6 months and post-treatment	Sustained reduction in chronic alcohol and/or cannabis use and increased abstinence Sustained improvement in psychological health Positive education, training, employment and accommodation outcomes	Moderate
Murphy (2011)	Case study 2007–2011	Koori youth residential AOD rehabilitation (Healing Service): Primary health counselling/mentoring, group work, aftercare, links to key adults in Koori community Average length of stay: 69 days	Sample size: 7 case studies, number of participants interviewed not specified	Koori residential program participant 15–20 years Problems relating to AOD use	15–20 years (68%m)	7 case studies based on oral story-telling and observations Interviews with young people, staff and stakeholders	Increased safety and pride in cultural background Improvements in communicating with adults, emotional regulation and relationships Appropriate aftercare essential	Low
Norberg et al. (2013)	RCT (parallel group) Year not stated	Brief CBT and motivational interviewing Immediate treatment (IT) group and a 3-month delayed treatment control (DTC) group Length of treatment: 3 sessions over 3 weeks (1 session per week)	Sample size: 33 IT (n = 18) DTC (n = 11)	14–30 years old ≥ weekly cannabis use in month prior to assessment Diagnosis of cannabis dependence English speaking	14–30 years (56.7%m)	Numerous clinical measures and scales for substance use and psychological distress	No significant improvements in the IT group compared with the DTC group for any of the outcome measures	High
Simpson et al. (2010)	Before-after study with no control group 2007–2009	Counselling clients of youth cannabis service received a median number of 3 treatment sessions over the course of a 2-year period	Sample size: 50 (counselling clients)	16–25 years cannabis dependence counselling clients	16–25 years (80%m)	Service outcome statistics dataset and the National Minimum Data Set for the years 2007–2009 (for comparison)	Significant decrease in frequency and amount of cannabis use Significant increase in life satisfaction	Low
Tait et al. (2010)	Systematic review and meta-analysis 2009	Internet-based attitudinal change and substance reduction interventions for young people with problematic substance use	16 studies	RCT Published in English Study outcome included a measure of consumption of the target substance	*Across studies:* Participant age range and %m not stated for all studies	Search of 3 electronic databases and reference lists	Web-based interventions targeting alcohol use by young adults appear effective in reducing alcohol problems in current drinkers	Low
Tait et al. (2016)	RCT (parallel group) 1999–2002	RCT conducted across 4 public hospital emergency departments (ED) in Perth. Comparison of treatment costs following an AOD-related presentation to ED. Usual care compared with brief counselling, advice and referral to link adolescents with external AOD services Length of treatment: 1 × 1 h session + referral and follow-up phone call	Sample size: 127 Control (n = 67) Intervention (n = 60)	12–19 years AOD-related presentation to ED	12–19 years (%m not stated)	Health data linkage used for 10 years following the brief intervention	Those who received the intervention had lower costs of ED mental health AOD presentations Injecting drug use was a significant baseline predictor of subsequent costs	Low
Wachtel et al. (2010)	Literature review 1998–2008	Brief clinical interventions including motivational interviewing and harm minimisation aimed at reducing alcohol misuse and binge drinking	14 RCTs	RCT Published in English Brief intervention studies specific to alcohol reduction Participant age: 12– 25 years	*Across studies:* Participant age range and %m not stated for all studies	6 online databases and reference lists	No single intervention recommended due to confounding evidence	Moderate

### Individual

Individual interventions included counselling and mentoring. The combination of cognitive behaviour therapy (CBT), motivational interviewing (MI) and mindfulness delivered over 10 sessions was found to result in sustained improvements in anxiety and depression, coping skills and functioning with decreased substance use [29]. The addition of CBT and MI to standard care of case management and brief MI (BMI) resulted in significant reductions in depression and cannabis use with sustained increases in motivation and socialisation [30]. The combined three strategies of BMI, coping skills training and psycho-education with the addition of personalised assessment feedback were found to be more effective than a brief assessment and general information only in reducing alcohol and substance use intake and psychological distress [31]. A range of focused psychological strategies over 5 sessions resulted in reduced psychological distress compared with other treatment and no treatment [23]. A median number of 3 counselling sessions delivered over a 2-year period resulted in a decrease in the frequency and amount of cannabis use and increased life satisfaction [36].

### Family

Family-based interventions included family, therapy and family counselling. A systematic review of family-based interventions for alcohol misuse and alcohol-related harm in Indigenous communities found that family-based interventions led to positive effects in individual and family functioning and coping and decreased alcohol consumption [28].

### Organisational

One effective organisational intervention was continuing care and community integration in a hospital setting. A study in four emergency departments of usual care, compared with brief advice and a referral linkage to an external alcohol and other drug (AOD) service, found a reduced number of mental health and AOD presentations in emergency departments (ED) for non-injecting drug use [39].

### Multiple interventions

Some studies had multiple interventions including combinations of individual and family work, education and training, recreation, peer groups, community and cultural engagement including mentoring. Two studies in residential programs that provided individual and family counselling, vocational education and training and recreation activities found sustained outcomes for decreased substance use, improved mental health and enhanced interpersonal relationships [20, 25]. Bush adventure therapy combined with counselling and continuing care resulted in reduced substance use and self-harm, and improvements in wellbeing [22]. An observational study in a residential therapeutic community found individually tailored recreation, art therapy and vocational education to be more effective than journaling and encounter groups [35]. Validating relationships with family, peers and service providers was found to be the key to the success of a service that provided a range of interventions in residential, day and outreach programs [40]. A study in a residential Koori youth rehabilitation (healing) service providing individual, family, group and community aftercare services found these interventions resulted in increased safety, pride in culture, and improvements in relationships and emotional regulation. Appropriate aftercare was considered essential [26]. A literature review found successful AOD interventions targeted at Aboriginal young people included flexibility, strong engagement with culture and community and addressed individual, community and systemic issues [21].

## Limitations

As mentioned earlier, this scoping review was limited according to study location and timeframe. The reliability of the findings is limited due to the low quality of studies, in terms of risk of bias and the study of interventions provided in conjunction with other interventions that were not evaluated at the same time. The non-blinding of participants in some of the controlled experimental studies [23, 30–32, 37, 39] could have biased participant responses. Participants may have felt pressured to respond positively to remain in the treatment program, not lose privileges and maintain positive relationships with staff.

## Discussion

This scoping review identified a range of individual, family, organisational and multiple combined interventions for working with adolescents using substances. The findings reveal the effectiveness of particular groupings (not all) of interventions in residential and after-care settings. CBT and MI were found to be effective together [37] and when combined with mindfulness [29], or psychoeducation, coping skills training and personalised assessment feedback [31] or case management [30]. Three of these were brief interventions (BI) between 2–3 sessions [29, 31, 37]. These findings support other research on the effectiveness of BI for this population group [41]. The effectiveness of individual CBT and MI interventions is well documented in evidence-based international research [42].

The study findings highlight important issues related to interventions with no evidence-base such as journaling, recreation and adventure activities, and encounter groups with adolescents. An intervention warranting further investigation is journaling. For instance, journaling was in a multiple intervention study [20] that produced significant outcomes. However, in another study journaling received a strong negative reaction from some adolescents [35]. Peer feedback encounter groups also warrant closer investigation as this same study found that a group with adolescents providing direct feedback to each other resulted in social ostracization [35].

Encounter groups are frequently used in adult residential substance use services. However, the use of these groups with adolescents requires further scrutiny due to adolescents developmental needs related to reasoning capacity, identity formation and relationships [3]. Other studies found groupwork to be beneficial [22, 26, 36]. The types of groups varied and were provided as part of a package of interventions. Building positive and affirming relationships with adolescents was considered essential for effective treatment outcomes. Notions of success were variable across the studies with improvement considered a success regardless of whether or not the adolescent completed the treatment program. The importance of continuing care was highlighted with some adolescents having multiple separate periods of engagement with a service/s over time.

### Future research, practice and policy considerations

Further research and evaluation is required on all therapeutic interventions currently used with young people using substances. In particular, interventions that do not have an evidence-base for use with adolescents such as encounter groups and journaling require closer scrutiny. It is recommended that intervention costings information be provided in future studies to inform public health resource allocation decision-making.

## Conclusion

The main finding of this scoping review is that there is currently no evidence base for some of the therapeutic interventions frequently provided to adolescents by alcohol and other drugs service providers. There is no evidence that services such as encounter groups, that are used widely in adult alcohol and other drugs services, are directly transferable to the adolescent population. This is particularly so due to the unique developmental stage and associated tasks at adolescence disrupted by substance use. Nonetheless, the studies examined in this scoping review, report a number of combined interventions that are considered effective. The lack of evidence for all of these interventions, when delivered in combination, hinders the ability to assess intervention specific benefits.
